# Supplementary material for: Isolated Cognitive Decline in Neurologically Stable Patients with Multiple Sclerosis
Source: Diagnostics (Basel). 2021 Mar 7;11(3):464. doi: 10.3390/diagnostics11030464 (PMC7999620; doi:10.3390/diagnostics11030464)
Supplement: Supplementary file 1 [file diagnostics-11-00464-s001.zip › supplementary tables.pdf]

Supplementary Tables

Table S1: Binary logistic regression results, GROUP 1 and GROUP 2 membership as a dependent variable (Baseline – Month 12)

	B (SE)	Odds Ratio	95% CI	p-value
Intercept	-21.067 (9.822)	7.09^{-10}	3.09^{-18}-0.163	0.032*
MS Duration	0.033 (0.027)	1.033	0.981-1.089	0.218
Baseline EDSS	0.336 (0.159)	1.399	1.026-1.909	0.034*
Baseline BPF	0.210 (0.115)	1.233	0.984-1.545	0.069
Baseline T1Lesion Volume (Square Root)	0.487 (0.360)	1.627	0.803-3.295	0.176
Baseline CCF	-4.125(4.170)	0.016	4.56^{-6}-57.311	0.323
Baseline SDMT Z-score	0.206 (0.154)	1.229	0.909-1.662	0.181

Legend: BPF = Brain parenchymal fraction, CCF = corpus callosum fraction; R^2 = 0.022 (Cox & Snell), 0.062 (Nagelkerke), 0.051 (McFadden). Model χ^2 = 13.8(6), p= 0.032.

Predictors selected from the pool of variables showing meaningful between-group differences (see Table 1), based on results of Omnibus Likelihood Ration Test (p<0.7) and on VIF statistics preventing collinearity issues.

* p < 0.05, ** p < 0.01, *** p < 0.001

Table S2: [Confirmatory Analysis, Month 12 – Month 24] Binary logistic regression results, GROUP 1 and GROUP 2 membership as a dependent variable (Month 12 – Month 24)

	B (SE)	Odds Ratio	95% CI	p-value
Intercept	26.354 (9.479)	2.79^{11}	2383.6-3.27^{19}	0.005**
MS Duration	-0.037 (0.033)	0.964	0.903-1.029	0.274
M12 EDSS	0.208 (0.164)	1.231	0.893-1.696	0.204
M12 BPF	-0.338 (0.114)	0.713	0.570-0.893	0.003**
M12 T1 Lesion Volume (Square Root)	0.524 (0.340)	1.690	0.867-3.292	0.123
M12 CCF	-4.879 (5.226)	0.008	2.71^{-7}-213.786	0.351
M12 SDMT Z-score	0.456 (0.160)	1.578	1.154-2.157	0.004**

Legend: M12 = Month 12, BPF = Brain parenchymal fraction, CCF = corpus callosum fraction; $R^2 = 0.055$ (Cox & Snell), 0.169 (Nagelkerke), 0.143 (McFadden). Model $\chi^2 = 31.9(6)$, $p < 0.001$.

For comparability, similar predictors as in the main analysis were selected (see Supplementary Table 4).

* $p < 0.05$, ** $p < 0.01$, *** $p < 0.001$

Table S3: Change of basic sample characteristics over 12 months (Baseline – Month 12)

	N Total	All Groups	GROUP 1	GROUP 2	GROUP 3	GROUP 4	Sig.	Effect Size
**Neurological**								
EDSS Change†	1091	0 (0 – 0)	0 (0 – 0)	0 (0 – 0)	0 (0 – 0.5)	0.5 (0 – 0.5)	N/A‡	N/A‡
≥1 Relapse Baseline-Month 12 (%)	1091	266 (24.4%)	0 (0%)	0 (0%)	250 (74.0%)	16 (61.5%)	N/A‡	N/A‡
Treatment Escalation (%)	1091	143 (13.1%)	63 (9.2%)	6 (13.6%)	70 (20.7%)	4 (15.4%)	**< 0.001**[3]	0.155[4]
**Neuropsychological**								
SDMT Z-Score Change	1091	0.14±0.78	0.24±0.67	-1.57±0.87	0.25±0.61	-1.17±0.49	N/A‡	N/A‡
BDI Total Score Change	1091	-0.19±5.43	-0.34±5.01	0.36±6.96	0.07±6.00	-0.65±5.71	0.396[1]	0.003[2]
BDI Baseline – M12 Worsening (%)	1091	46 (4.2%)	20 (2.9%)	3 (6.8%)	22 (6.5%)	1 (3.8%)	0.047[3]	0.085[4]
BDI Baseline – M12 Depressed (%)	1091	431 (39.5%)	232 (34.0%)	24 (54.5%)	163 (48.2%)	12 (46.2%)	**< 0.001**[3]	0.149[4]
**Radiological**								
BPF Change (%)	763	-0.07±0.77	-0.01±0.63	-0.29±0.65	-0.11±0.98	-0.60±1.03	**0.002**[1]	0.019[2]
CC Change (%)	762	-1.65±2.37	-1.61±2.26	-1.92±2.68	-1.70±2.54	-1.70±2.74	0.690[1]	0.002[2]
T1 LV Change (ml)	749	0.05±0.45	0.05±0.41	0.01±0.27	0.06±0.48	0.18±1.06	0.973[1]	0.000[2]
T2 LV Change (ml)	749	0.15±1.23	0.08±0.97	-0.01±1.17	0.21±1.37	1.42±3.44	0.398[1]	0.004[2]

Legend: Group 1 = Neurologically & Cognitively Stable, Group 2 = Neurologically Stable but Cognitively Worsening, Group 3 = Neurologically Worsening but Cognitively Stable, Group 4 = Neurologically and Cognitively Worsening, BPF = Brain parenchymal fraction, CC = corpus callosum, T1 LV = T1 Lesion Volume, T2 LV = T2 Lesion Volume, M12 = Month 12; [1]Kruskal-Wallis H test, [2]ε^2, [3]χ^2-test, [4]Cramer's-V; Differences losing significance after BH procedure are not highlighted. Unless otherwise indicated, mean±SD reported.

‡ The difference was not statistically tested because it stems from the group definition per se

† Reported median and interquartile range

Table S4: [Confirmatory Analysis, Month 12 – Month 24] Basic sample characteristics at Month 12

**Characteristics**	N Total	All Groups	GROUP 1	GROUP 2	GROUP 3	GROUP 4	Sig.	Effect Size
**Demographic**								
Number of patients (%)	1060	1060 (100%)	692 (65.3%)	50 (4.7%)	291 (27.5%)	27 (2.6%)	N/A	N/A
Females (%)	1060	733 (69.2%)	459 (66.3%)	38 (76.0%)	215 (73.9%)	21 (77.8%)	0.053[1]	0.085[2]
Age	1060	38.5±9.1	38.4±9.1	42.1±10.0	37.8±8.7	40.3±7.6	**0.017[3]**	0.010[4]
Education	1060	14.7±3.0	14.7±3.0	15.0±3.1	14.6±3.0	14.3±3.1	0.757[3]	0.001[4]
**Neurological**								
Disease Duration	1060	10.0±7.3	9.5±7.2	12.8±8.1	10.3±7.2	12.7±7.6	**0.002[3]**	0.014[4]
EDSS†	1060	2.0 (1.5 – 3.5)	2.0 (1.5 – 3.5)	3.25 (2.0 – 4.0)	2.5 (1.5 – 4.0)	3.5 (2.0 – 4.0)	**< 0.001[3]**	0.027[4]
DMT (%)	1060						0.585[1]	0.047[2]
High Efficacy		276 (26.0%)	171 (24.7%)	14 (28.0%)	80 (27.5%)	11 (40.7%)		
Low Efficacy		657 (62.0%)	439 (63.4%)	29 (58.0%)	175 (60.1%)	14 (51.9%)		
**Neuropsychological**								
Cogn. Impaired ‡ (%)	1060	325 (30.7%)	187 (27.0%)	20 (40.0%)	105 (36.1%)	13 (48.1%)	**0.003[1]**	0.116[2]
SDMT Z-Score	1060	-0.87±1.41	-0.79±1.33	-0.90±1.98	-1.06±1.43	-0.90±1.93	0.058[3]	0.007[4]
BDI Total Score	1060	7.4±7.4	6.8±7.1	7.6±7.1	9.0±7.7	6.9±8.4	**< 0.001[3]**	0.020[4]
**Radiological**								
BPF (%)	796	85.1±2.3	85.2±2.19	83.2±2.56	85.0±2.45	84.5±2.50	**< 0.001[3]**	0.023[4]
CCF (%)	794	0.30±0.05	0.30±0.05	0.26±0.05	0.30±0.05	0.29±0.05	**0.003[5]**	0.015[6]
T1 Lesion Volume (ml)	796	1.62±2.09	1.46±1.93	3.18±3.40	1.79±2.19	2.06±1.89	**0.002[3]**	0.019[4]
T2 Lesion Volume (ml)	796	4.84±7.65	4.25±6.99	12.6±15.2	5.27±7.35	5.20±6.36	**< 0.001[3]**	0.021[4]

Legend: Group 1 = Neurologically & Cognitively Stable, Group 2 = Neurologically Stable but Cognitively Worsening, Group 3 = Neurologically Worsening but Cognitively Stable, Group 4 = Neurologically and Cognitively Worsening, DMT = Disease modifying treatment, Cogn. Impaired = Cognitively Impaired BPF = Brain parenchymal fraction, CCF = corpus callosum fraction; [1]χ^2-test, [2]Cramer's-V, [3]Kruskal-Wallis H test, [4]ε^2, [5]Fischer's One-Way ANOVA, [6]η^2; Differences losing significance after BH procedure are not highlighted. Unless otherwise indicated, mean±SD reported.

‡ Cognitive impairment defined as SDMT below the benchmark of -1.5 SD, using norms adjusted for age and education.

† Reported median and interquartile range

Table S5: [Confirmatory Analysis, Month 12 – Month 24] Change of basic sample characteristics over 12 months (Month 12 – Month 24)

	N Total	All Groups	GROUP 1	GROUP 2	GROUP 3	GROUP 4	Sig.	Effect Size
**Neurological**								
EDSS Change†	1060	0 (0 – 0)	0 (0 – 0)	0 (0 – 0)	0 (0 – 0.5)	0.5 (0 – 0.5)	N/A‡	N/A‡
≥1 Relapse M12 – M24 (%)	1060	247 (23.3%)	0 (0%)	0 (0%)	227 (78.0%)	20 (74.1%)	N/A‡	N/A‡
Treatment Escalation (%)	1060	80 (7.5%)	31 (4.5%)	2 (4.0%)	44 (15.1%)	3 (11.1%)	**< 0.001**[3]	0.181[4]
**Neuropsychological**								
SDMT Z-Score Change	1060	0.05±0.77	0.17±0.64	-1.40±0.70	0.14±0.65	-1.51±0.51	N/A‡	N/A‡
BDI Total Score Change	1057	-0.06±5.33	-0.10±4.95	-0.80±5.54	-0.06±6.12	2.30±5.18	0.081[1]	0.006[2]
BDI M12 – M24 Worsening (%)	1057	42 (4.0%)	24 (3.5%)	2 (4.1%)	13 (4.5%)	3 (11.1%)	0.235[3]	0.064[4]
BDI M12 – M24 Depressed (%)	1050	405 (38.2%)	231 (33.4%)	19 (38.0%)	144 (49.5%)	11 (40.7%)	**< 0.001**[3]	0.146[4]
**Radiological**								
BPF Change (%)	635	-0.24±0.60	-0.20±0.60	-0.22±0.47	-0.31±0.60	-0.37±0.61	0.455[1]	0.004[2]
CC Change (%)	623	-0.67±1.92	-0.55±1.90	-1.48±2.10	-0.80±1.97	-1.14±1.22	0.050[1]	0.013[2]
T1 Lesion Volume Change (ml)	635	0.18±0.37	0.15±0.28	0.42±0.48	0.21±0.48	0.34±0.65	0.060[1]	0.012[2]
T2 Lesion Volume Change (ml)	635	0.42±1.43	0.29±1.05	1.68±2.79	0.55±1.86	0.67±1.37	**0.006**[1]	0.019[2]

Legend: Group 1 = Neurologically & Cognitively Stable, Group 2 = Neurologically Stable but Cognitively Worsening, Group 3 = Neurologically Worsening but Cognitively Stable, Group 4 = Neurologically and Cognitively Worsening, BPF = Brain parenchymal fraction, CC = corpus callosum, M12 = Month 12, M24 = Month 24; [1]Kruskal-Wallis H test, [2]ε^2, [3]χ^2-test, [4]Cramer's-V; Differences losing significance after BH procedure are not highlighted. Unless otherwise indicated, mean±SD reported.

‡ The difference was not statistically tested because it stems from the group definition per se

† Reported median and interquartile range

Table S6: [Confirmatory Analysis, Month 12 – Month 24] Post-Hoc pairwise comparison of selected subgroups at Month 12

	GROUP 1 vs. GROUP 2		GROUP 1 vs. GROUP 4		GROUP 2 vs. GROUP 4		GROUP 3 vs. GROUP 4	
	Mean Difference / W	Sig.	Mean Difference / W	Sig.	Mean Difference / W	Sig.	Mean Difference / W	Sig.
Age	3.79	**0.037**	1.74	0.609	-1.34	0.781	2.18	0.414
Disease Duration	4.02	**0.023**	3.34	0.085	-0.14	1.000	2.284	0.370
EDSS	4.85	**0.003**	3.26	0.097	-0.48	0.987	1.07	0.874
BDI Total Score	1.56	0.688	-0.31	0.996	-1.23	0.821	-2.47	0.299
BPF	-5.77	**< 0.001**	-2.05	0.467	1.90	0.535	-1.62	0.662
CCF	0.03	**0.005**	0.02	0.521	-0.01	0.766	0.01	0.890
T1 Lesion Volume	4.69	**0.005**	2.43	0.315	-1.43	0.745	1.40	0.757
T2 Lesion Volume	5.21	**0.001**	2.09	0.452	-2.35	0.346	1.03	0.886
T2 Lesion Volume Change	4.09	**0.020**	0.78	0.947	-1.87	0.548	-0.23	0.999

Legend: Group 1 = Neurologically & Cognitively Stable, Group 2 = Neurologically Stable but Cognitively Worsening, Group 3 = Neurologically Worsening but Cognitively Stable, Group 4 = Neurologically and Cognitively Worsening, BPF = Brain parenchymal fraction, CCF = corpus callosum fraction; [1]Dwass-Steel-Critchlow-Fligner, [2]Tukey
