# Supplementary figures and images for: Isolated Cognitive Decline in Neurologically Stable Patients with Multiple Sclerosis
Source: Diagnostics (Basel). 2021 Mar 7;11(3):464. doi: 10.3390/diagnostics11030464 (PMC7999620; doi:10.3390/diagnostics11030464)

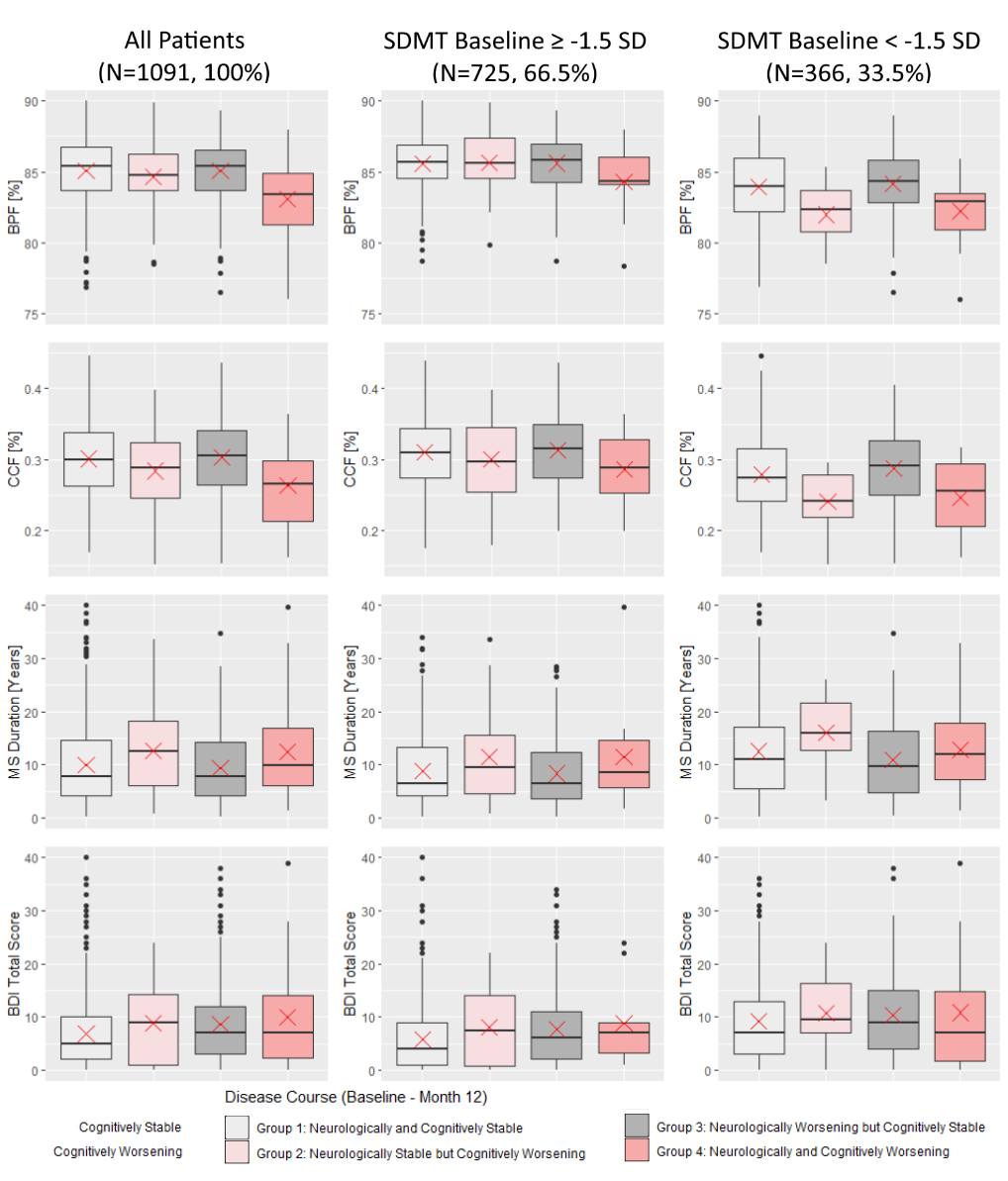

Supplement: Supplementary file 1 [file diagnostics-11-00464-s001.zip › Figure_S1.png]

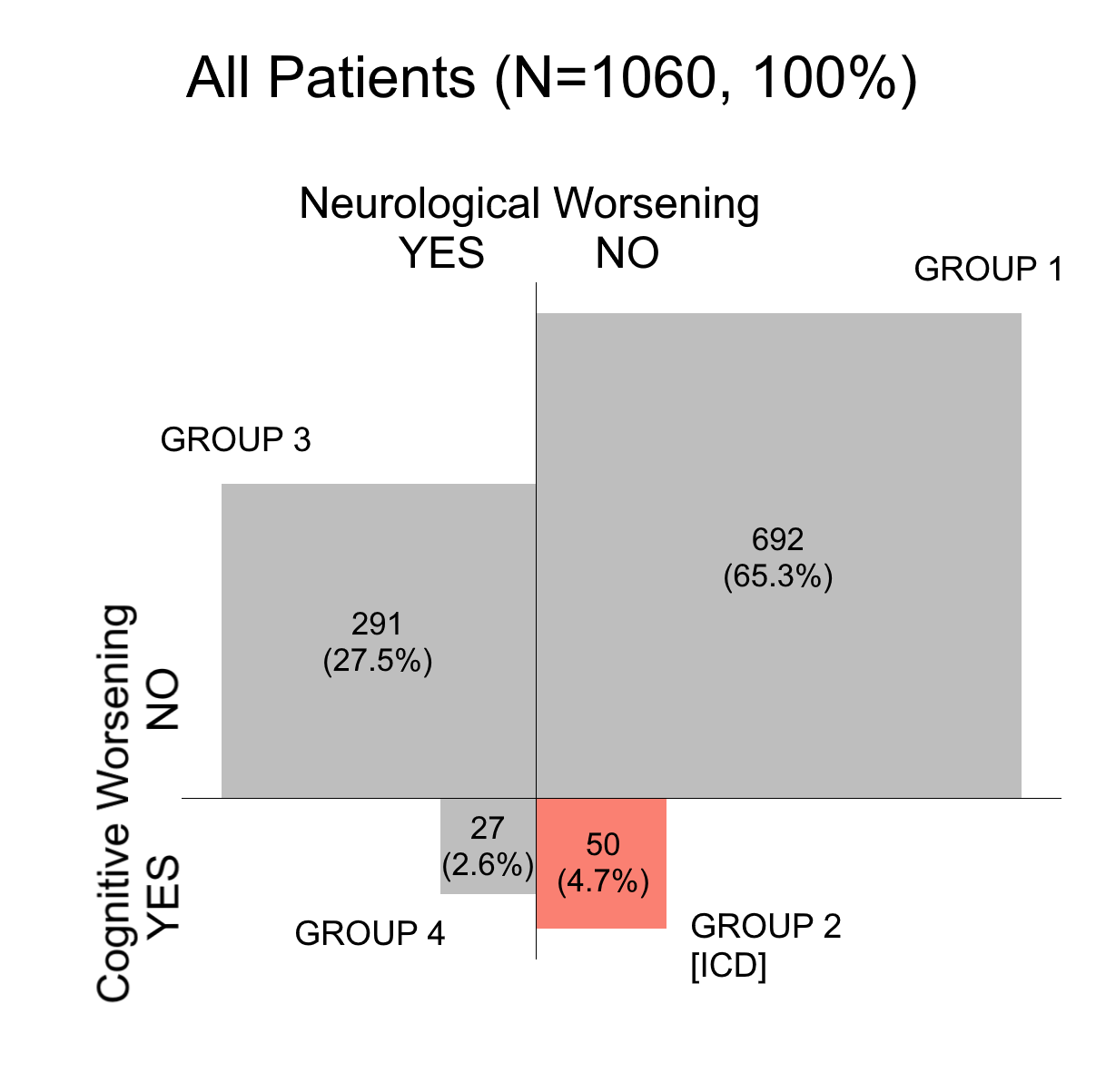

Supplement: Supplementary file 1 [file diagnostics-11-00464-s001.zip › Figure_S2.png]

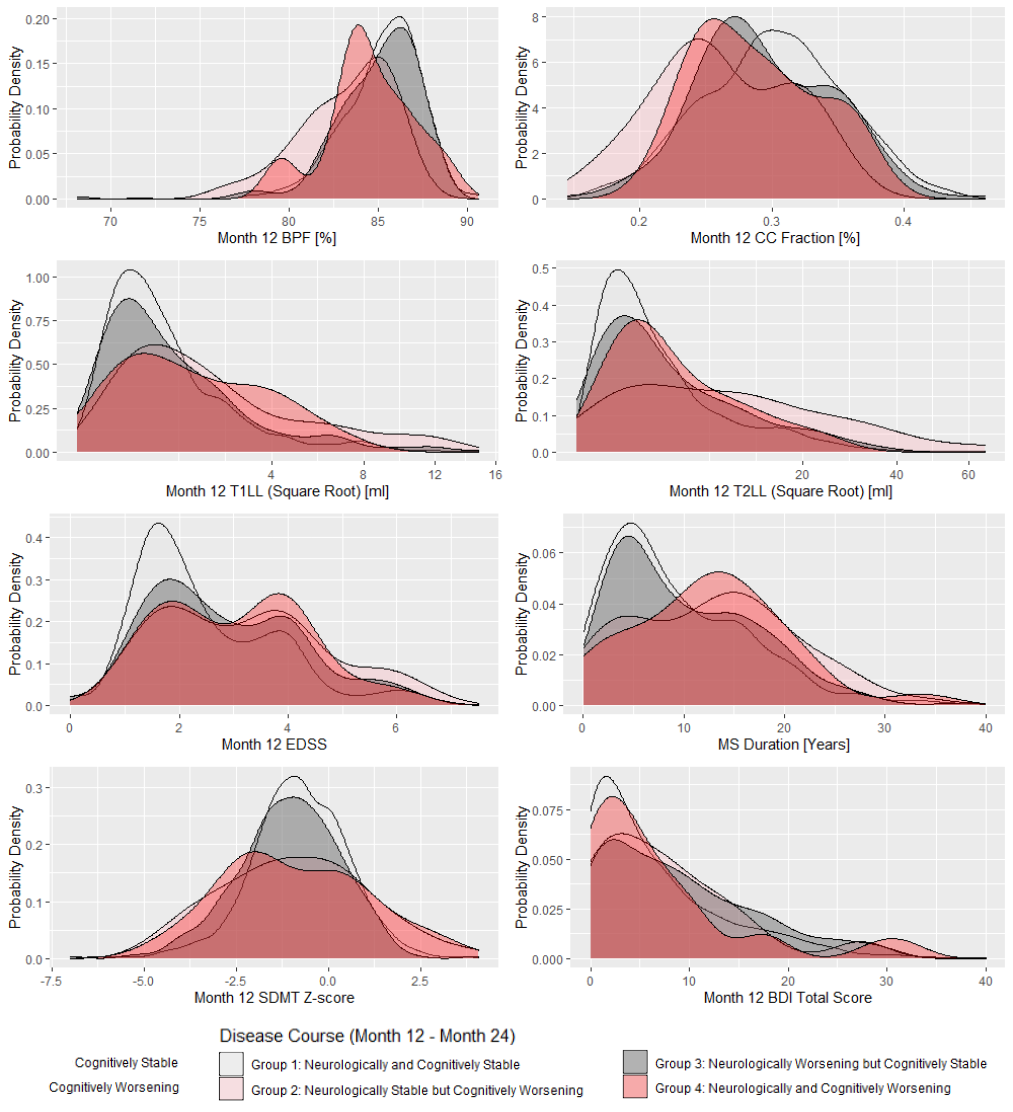

Supplement: Supplementary file 1 [file diagnostics-11-00464-s001.zip › Figure_S3.png]
